# Imaging of peritoneal metastases of ovarian and colorectal cancer: joint recommendations of ESGAR, ESUR, PSOGI, and EANM

**DOI:** 10.1007/s00330-024-11124-5

**Published:** 2024-11-05

**Authors:** Vincent Vandecaveye, Pascal Rousset, Stephanie Nougaret, Artem Stepanyan, Milagros Otero-Garcia, Olivera Nikolić, Maira Hameed, Karolien Goffin, Ignace H. J. de Hingh, Max J. Lahaye, for the European Society of Gastrointestinal and Abdominal Radiology (ESGAR), Vincent Vandecaveye, Pascal Rousset, Stephanie Nougaret, Maira Hameed, Max J. Lahaye, European Society of Urogenital Radiology (ESUR), Stephanie Nougaret, Milagros Otero-Garcia, Olivera Nikolić, Peritoneal Surface Oncology Group International (PSOGI), Max J. Lahaye, Ignace H. J. de Hingh, European Association of Nuclear Medicine (EANM), Karolien Goffin

**Affiliations:** 1 Department of Radiology, University Hospitals Leuven, Leuven, Belgium; 2 Division of Translational MRI, Department of Imaging and Pathology, KU Leuven, 3000 Leuven, Belgium; 3 Department of Radiology, Hospices Civils de Lyon, Lyon Sud University Hospital, Lyon 1 Claude Bernard University, 69495 Pierre Bénite, France; 4 Department of Radiology, Montpellier Cancer Institute, Montpellier, France; 5 PINKCC Lab, U1194, IRCM, Montpellier, France; 6 Gynecologic Oncology Service, NAIRI Medical Center, 0015 Yerevan, Armenia; 7 Department of Radiology, University Hospital Vigo (Hospital Alvaro Cunqueiro), Instituto de Investigación Sanitaria Galicia Sur (IISGS), 36213 Vigo, Spain; 8 University of Novi Sad, Faculty of Medicine, Center for Radiology, University Clinical Center of Vojvodina, 21000 Novi Sad, Serbia; 9 University College London Hospitals NHS Foundation Trust, London, UK; 10 University College London Centre for Medical Imaging, Charles Bell House, W1W 7TS London, UK; 11 Nuclear Medicine, University Hospital Leuven, Leuven, Belgium; 12 Nuclear Medicine & Molecular Imaging, Department of Imaging and Pathology, KU Leuven, Leuven, Belgium; 13 Catharina Cancer Institute, Eindhoven, the Netherlands; 14 Department of Epidemiology, GROW Research Institute for Oncology and Reproduction, Maastricht University Medical Center+, Maastricht, the Netherlands; 15 Netherlands Cancer Institute, Department of Radiology, 1066 CX Amsterdam, The Netherlands; 16 GROW Research Institute for Oncology and Reproduction, Maastricht University Medical Center+, Maastricht, the Netherlands

**Keywords:** MRI, CT, Peritoneal metastases, Colorectal cancer, Ovarian cancer

## Abstract

**Objectives:**

Diagnostic imaging of peritoneal metastases in ovarian and colorectal cancer remains pivotal in selecting the most appropriate treatment and balancing clinical benefit with treatment-related morbidity and mortality. To address the challenges related to diagnostic imaging and detecting and reporting peritoneal metastatic spread, a joint guideline was created by the European Society of Gastrointestinal and Abdominal Radiology (ESGAR), European Society of Urogenital Radiology (ESUR), Peritoneal Surface Oncology Group International (PSOGI), and European Association of Nuclear Medicine (EANM).

**Methods:**

A targeted literature search was performed and consensus recommendations were proposed using Delphi questionnaires and a five-point Likert scale.

**Results:**

A total of three Delphi rounds were performed. Consensus was reached on the position of diagnostic imaging for assessment of operability, treatment response monitoring, and follow-up of peritoneal metastases, optimal imaging modality and their technical imaging requirements depending on the indication and how to optimise communication of imaging results by the report and multidisciplinary board discussion. The complete list of recommendations is provided.

**Conclusion:**

These expert consensus statements aim to guide appropriate indications, acquisition, interpretation, and reporting of imaging for operability assessment, treatment response monitoring, and follow-up of peritoneal metastases in ovarian and colorectal cancer patients.

**Key Points:**

***Question*** *Staging peritoneal metastases (PM) helps to guide clinical decision-making for colorectal and ovarian cancer patients. How can we optimise the use of imaging techniques to assess PM?*

***Findings*** *Imaging plays a crucial role in the detection, operability assessment, treatment response monitoring, and follow-up of peritoneal metastases in colorectal and ovarian cancer patients.*

***Clinical relevance*** *These expert consensus statements aim to guide appropriate indication, acquisition, interpretation, and reporting of imaging for operability assessment, treatment response monitoring, and follow-up of peritoneal metastases in ovarian and colorectal cancer patients.*

## Main recommendations

- Radiological imaging plays a crucial role in the detection, operability assessment, treatment response monitoring, and follow-up of peritoneal metastases in colorectal and ovarian cancer patients. 100% agreement, low–moderate quality evidence.
- Although CT tends to underestimate the extent of peritoneal metastases, CT can be used for the initial assessment at the time of diagnosis, treatment response monitoring, and suspicion of recurrence of peritoneal metastases in colorectal and ovarian cancer patients due to its widespread availability. 100% agreement, low–moderate quality evidence.
- CT is the most accessible technique in daily clinical routine and can help to identify patients with extraperitoneal or widespread peritoneal disease that could be excluded from upfront surgery. Due to the low accuracy for detecting small or isodense peritoneal metastases, CT shows suboptimal results in selecting patients in which complete cytoreductive surgery is feasible. 100% agreement, low–moderate quality evidence.
- MRI is currently the most accurate imaging modality to assess the extent of peritoneal metastases. It can play a role in selecting colorectal and ovarian cancer patients for cytoreductive surgery at primary diagnosis, after neoadjuvant chemotherapy or tumour recurrence. 100% agreement, low–moderate quality evidence.
- FDG PET/CT is not accurate enough for pre-operative staging, missing small lesions, as CT and hypometabolic (e.g., mucinous) metastases. Still, FDG PET/CT can help detect other extraperitoneal metastases missed on prior imaging. 100% agreement, low–moderate quality evidence.
- Whole-body MRI and FDG PET/CT can be a problem-solver in colorectal and ovarian cancer patients suspected of peritoneal metastases/recurrence. Both modalities can help to detect other extraperitoneal metastases missed on prior imaging. 100% agreement, low–moderate quality evidence.
- An abdominal CT for peritoneal metastases consists of 1/1.5 mm section images with 3 mm axial and coronal or/and sagittal reconstructions in the portal venous phase. Positive or negative oral contrast agents are highly recommended but can be omitted based on local preferences. 85% agreement, low–moderate quality evidence.
- An abdominal or whole-body 3-T MRI for peritoneal metastases consists of axial T2 (slice thickness ≤ 4 mm), STIR-DWI (b50 and b1000 s/mm^2^), and post-contrast (after 3–5 min) T1 sequences in axial/coronal view. Axial pre-contrast T1 and coronal T2-weighted sequences are optional. One litre pineapple juice as a negative oral contrast and an intravenous antiperistatical agent is highly recommended but not obligatory. 85% agreement, low–moderate quality evidence.
- FDG PET/CT for peritoneal metastases should be performed according to the ‘EANM procedure guidelines for tumour imaging: version 2’. The combination with a CT of diagnostic quality, as described in statement 7, is highly recommended. 100% agreement, low–moderate quality evidence.
- There are no differences in FDG PET/CT, CT, or MR imaging protocols of the abdomen for peritoneal metastases between colorectal and ovarian cancer. 100% agreement, low–moderate quality evidence.
- The imaging report of colorectal cancer patients should include the extent of peritoneal metastases using the Peritoneal Cancer Index (PCI) combined with a qualitative description of the involvement of surgically critical sites and involved abdominal organs. Tools like PROMISE and PAUSE can be used to optimise and standardise the imaging report. 100% agreement, low–moderate quality evidence.
- The imaging report of ovarian cancer patients must include the extent of peritoneal metastases using one quantitative scoring method of local preference in combination with a qualitative description of the involvement of surgically critical sites and involved abdominal organs. Tools like PROMISE and PAUSE can be used to optimise and standardise the imaging report. 100% agreement, low–moderate quality evidence.
- Preferably, all colorectal and ovarian cancer patients with peritoneal metastases should be discussed in a multidisciplinary tumour board, including a radiologist with experience in imaging of peritoneal metastases. 100% agreement, low–moderate quality evidence.

## Introduction

Peritoneal metastases (PM) are common in ovarian and colorectal cancer patients. In colorectal cancer, PM occur in 6% of patients at initial diagnosis of the primary tumour and up to 21% as recurrent disease after surgery in pT4 patients [1, 2]. Due to the absence of symptoms of ovarian cancer in the early stages, approximately 80% of patients present with peritoneal disease. In both ovarian and colorectal cancer, PM is associated with an adverse prognosis [3, 4]. The presence and extent of PM have a major impact on patient management and treatment decisions. In colorectal cancer, the ‘peritoneal cancer index’ (PCI) is widely accepted as an accurate surgical tool to select those patients who might benefit from cytoreductive surgery ± HIPEC [5]. To determine the PCI, the abdomen is divided into nine regions and the small bowel into four additional regions. Each region receives a score of 0–3 based on the largest tumour size in that region. Total scores range from 0 to 39. This accurate assessment of the presence and full extent of PM is pivotal to aid in the decision to start treatment either by surgery or (neoadjuvant) chemotherapy and avoid undesirable open-close surgical procedures [6, 7]. In well-selected groups of patients with metastatic ovarian and colorectal cancer, 5-year survival rates of up to 50% are reported [8, 9].

Unfortunately, imaging of PM poses many challenges for radiologists and nuclear medicine physicians. Depiction of the total spread of PM and surgically critical lesions can be difficult due to the small size of the metastases or obscurement of metastases by ascites, nearby organs, or other structures.

This means that the total extent of PM can be easily underestimated, particularly in mesenteric and serosal bowel disease, requiring additional surgical staging procedures to assess operability [10]. This is an invasive procedure with a small risk of complications. In addition, diagnostic laparoscopies are often challenging and incomplete due to adhesions and confluent tumours obscuring the view of the peritoneal surface [11].

Additionally, communication of imaging findings regarding total disease extent and involvement of critical surgical sites require optimisation to effectively guide clinical and therapeutic decision-making.

Therefore, there is a clinical need to address these challenges of imaging PM. As this issue affects multiple clinical disciplines, representatives from several disciplines were included in compiling these multidisciplinary guidelines. These guidelines are also developed to familiarise radiologists with imaging techniques for PM and the clinical concepts of PM. These joint guidelines apply to all ovarian and colorectal cancer patients with a clinical suspicion of PM.

## Source and scope

These guidelines are recommendations developed by the European Society of Gastrointestinal and Abdominal Radiology (ESGAR), European Society of Urogenital Radiology (ESUR), Peritoneal Surface Oncology Group International (PSOGI), and European Association of Nuclear Medicine (EANM). A targeted literature search was performed to discover recent evidence concerning the imaging of peritoneal metastases in ovarian and colorectal cancer patients. The guidelines were formulated after careful consideration of the available literature by a group of international experts. The Grading of Recommendations Assessment, Development and Evaluation (GRADE) system was adopted to define the strength of recommendations and the quality of evidence.

## Methods

The ESGAR Research Committee appointed a chair (M.J.L.) to supervise guideline development; next to the chair, there were seven committee members. The chair selected three other ESGAR members based on their experience and authorships of prior relevant peer-reviewed indexed publications (V.V., P.R., S.N.). The European Society of Urogenital Radiology delivered two committee members (M.O.-G., O.N.). A representative of the following societies was also added to the guideline committee: Peritoneal Surface Oncology Group International (IdH) and the European Association of Nuclear Medicine (K.G.). The guideline committee complied with the ESGAR recommendations for guideline development principles and of the AGREE II instrument if applicable [12, 13].

A literature search was performed and included all relevant articles published until September 2021. A summary of the search strategy can be found in Appendix A. Two committee members (M.J.L. and V.V.), by consensus, composed a list of relevant articles based on the evaluation of all abstracts. This list was given to the guideline committee for approval. All members could add any additional suitable articles to be used as evidence. In addition, during the development of the guidelines, all relevant articles published until January 2023 could also be used as evidence.

Most literature was found for detecting peritoneal metastases with computed tomography (CT). Recently, more and more literature has been published concerning staging peritoneal metastases in colorectal and ovarian cancer using magnetic resonance imaging (MRI).

The chair devised draft Delphi questionnaires, which were further improved and approved by the guideline committee. The questions in the final approved questionnaire were used to formulate the consensus recommendations. All committee members scored their agreement with the recommendations using a five-point scale (1 = strongly disagree, 5 = strongly agree). Those statements achieving a score of 4 or 5 by at least 80% were accepted into the final set of consensus statements. Where consensus was not reached, another Delphi round was performed. In total, three Delphi rounds were required. The group were also asked to grade the level of evidence using the GRADE system.

The coordinating chairs sent the draft manuscript to all guideline committee members for improvements and approval. All participating societies reviewed the final manuscript for approval prior to submission for publication.

### Recommendations and statements

The recommendations are described below, and each point will be discussed separately.

Grades of evidence and agreement level of the group (in percentage) are included in brackets after each statement. Overall, the evidence is of moderate quality at best. This is mainly because the evidence is based primarily on single-centre prospective and retrospective studies and a lack of studies comparing different imaging techniques in the same patient cohort.

**1. Radiological imaging plays a crucial role in the detection, operability assessment, treatment response monitoring, and follow-up of peritoneal metastases in colorectal and ovarian cancer patients (100% agreement, low–moderate quality evidence)**.

Imaging aims to correctly identify the primary tumour or recurrence(s), malignant lymph nodes, PM, and other distant metastases. In addition, imaging can detect potential complications related to disease spread, such as hydronephrosis or small bowel obstruction [14]. In both ovarian and colorectal cancer, detecting PM and assessing total disease extent is essential to stage the patients and correctly select the optimal treatment strategy. In addition to surgical staging, imaging can detect disease spread in abdominal sites that are difficult to access by diagnostic laparoscopy, including the retro-hepatic area, the retroperitoneal spaces and the porta hepatis as well as distant metastatic disease sites [11]. Thus, imaging contributes to developing an individualised treatment strategy for patients suspected of PM. Despite the variable performance of different imaging modalities in assessing peritoneal disease spread, imaging allows for an initial less invasive and less costly diagnostic assessment than diagnostic laparoscopy.

**2. Although CT tends to underestimate the extent of peritoneal metastases, CT can be used for the initial assessment at the time of diagnosis, treatment response monitoring, and suspicion of recurrence of PM in colorectal and ovarian cancer patients due to its widespread availability. 100% agreement, low–moderate quality evidence**.

Although CT tends to underestimate the extent of disease in the abdomen, it provides a general overview of the disease and its potential complications in the abdomen. In a meta-analysis, CT had a pooled sensitivity of 68% and a pooled specificity of 84% for detecting PM [15]. However, the sensitivity of CT decreases rapidly with small lesions below 1 cm, particularly in the presence of ascites and in subdiaphragmatic, omental, mesenteric and serosal locations [16]. Chua et al [17] found that the accuracy of identifying peritoneal lesions with CT, regardless of size, ranged from 51 to 88% in abdominopelvic regions and from 21 to 25% in the small bowel regions. In lesions smaller than 5 mm, Koh et al found a sensitivity of only 11% [18]. This means that CT is not an accurate staging tool for PM. In ovarian cancer, for example, this underestimation can lead to unsuccessful or more complicated surgeries than anticipated in up to 40% of patients [19, 20]. Nevertheless, its widespread availability and moderate sensitivity for the overall detection of involved lymph nodes and metastases make CT an appropriate imaging tool for the initial assessment of ovarian and colorectal cancer at primary diagnosis and for detecting potential recurrences.

**3. CT is the most accessible technique in daily clinical routine and can help to identify patients with extraperitoneal or widespread peritoneal disease that could exclude upfront surgery. Due to the low accuracy for detecting small or isodense PM, CT plays a minimal role in selecting patients in which complete cytoreductive surgery is feasible. 100% agreement, low–moderate quality evidence**.

As mentioned above, CT will likely underestimate the extent of peritoneal metastases in colon and ovarian cancer.

For colorectal cancer, a meta-analysis showed that CT consistently underestimates the surgical PCI by 12–33% [21]. This was partly due to difficulties in calculating the PCI at CT and, in part, to limitations of CT for assessing different presentations of PM [22]. In ovarian cancer, studies have shown that CT is not reliable enough in predicting the completeness of debulking surgery, mainly attributable to difficulties in depicting small-sized metastases, particularly over the intestinal serosa and mesentery, but also due to the inability to accurately detect suprarenal or supradiaphragmatic lymphadenopathies or metastases to the liver hilum, coeliac trunk or pancreas.

While the inability to reliably assess occult disease at critical surgical sites and PCI limits its role in selecting patients for cytoreductive surgery, its widespread availability and robustness allow CT to be used for rapid initial assessment of colorectal or ovarian cancer patients. CT can depict potential disease features that would immediately affect treatment, including gross distant or (extra) peritoneal disease that would prohibit upfront surgery.

**4. STIR-DWI-MRI is currently the most accurate imaging modality to assess the extent of PM. It can play a role in selecting colorectal and ovarian cancer patients for cytoreductive surgery at primary diagnosis, after neoadjuvant chemotherapy or tumour recurrence. 100% agreement, low–moderate quality evidence**.

In several studies, including two meta-analyses, DWI-MRI was demonstrated to be highly sensitive and specific for detecting PM in gastrointestinal and ovarian cancer [15, 23]. Particularly in site-based analysis and for subcentimeter lesions, STIR-DWI-MRI shows high accuracy and outperforms CT and FDG-PET/CT for detecting PM. The high accuracy for site-based detection of PM enables DWI-MRI to assess potential surgically critical disease sites better. DWI-MRI allows better evaluation of the extent of serosal and mesenteric involvement compared to CT and FDG-PET/CT.

**Fig. 1:**
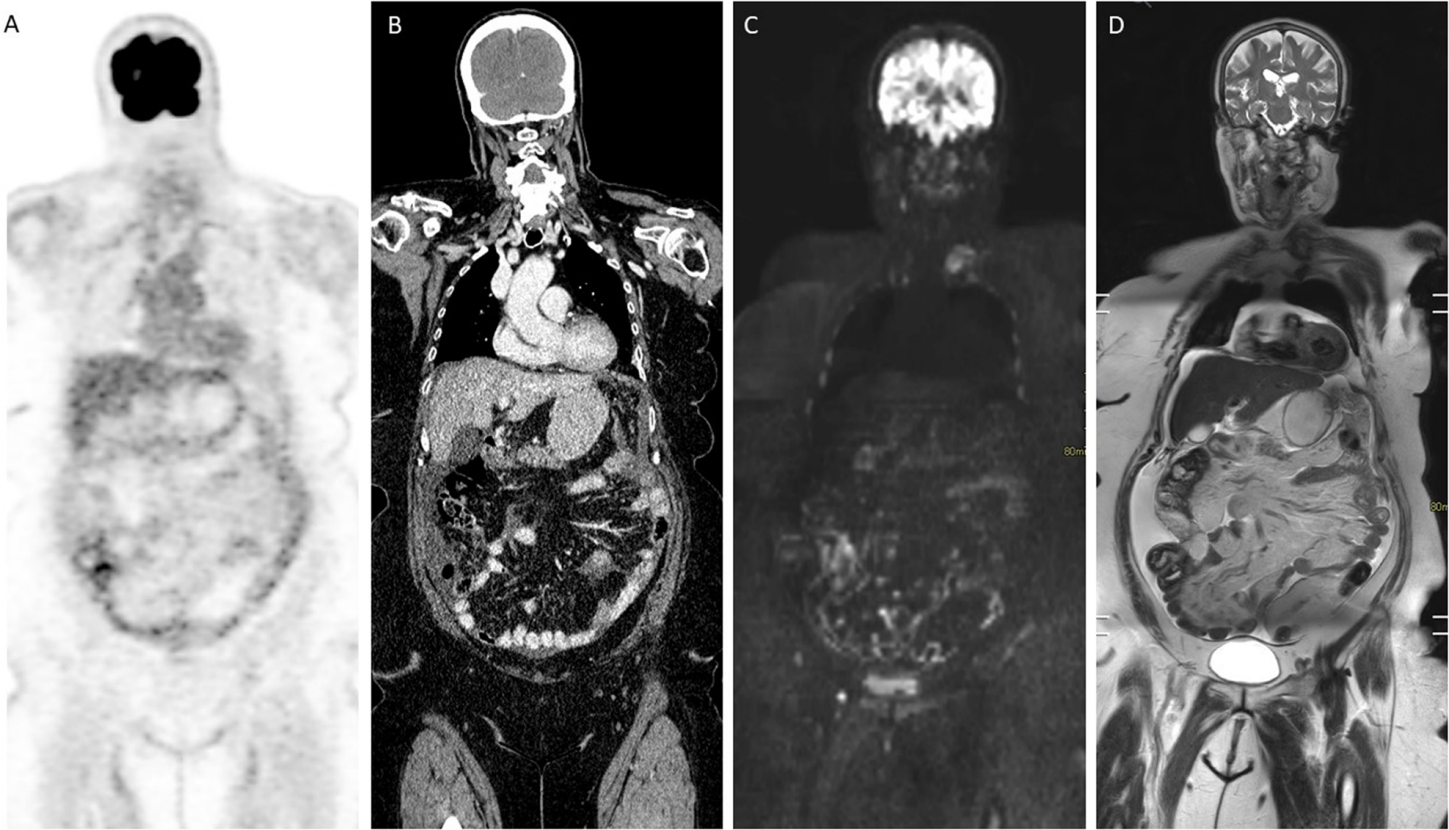
Patient presenting with a new diagnosis of fallopian tube cancer. **A**, **B** FDG-PET/CT imaging reveals faint diffuse uptake over the small bowel mesentery, accompanied by non-specific minimal mesenteric infiltration and ascites seen on CT. **C**, **D** Additional WB-DWI/MRI shows diffuse linear and micronodular infiltration of the small bowel mesentery and serosa, along with the presence of ascites on coronal reformatted DWI and T2-weighted sequences. Laparoscopy confirmed extensive presence of diffuse miliary metastases, with an average lesion size of 3 mm

Similarly, DWI-MRI shows higher accuracy for depicting potentially surgically critical upper abdominal disease involvement behind the portal vein, beyond the left and right main portal branches, involvement of the duodenum, stomach, pancreas and coeliac trunk, mesenteric root, and the main branch of the superior mesenteric artery. Additionally, DWI-MRI-based PCI has been shown to have the strongest correlation with surgical PCI [24]. The high accuracy of DWI-MRI for predicting disease at surgically critical disease sites and accurate prediction of surgical PCI has been shown in the primary diagnosis of PM in ovarian and colorectal cancer, recurrent PM in ovarian cancer and after neoadjuvant chemotherapy in colorectal cancer (see Fig. 1). This means that DWI-MRI can help in initial treatment planning by assessing disease load in anatomical locations that correlate with suboptimal cytoreduction or require specific surgical considerations to avoid perioperative morbidity or functional complications in case of potential extensive bowel resection.

**5. FDG PET/CT is not accurate enough for pre-operative staging, missing small lesions, as CT, and hypometabolic (e.g., mucinous) metastases. Still, FDG PET/CT can help detect other extraperitoneal metastases missed on prior imaging. Strong recommendation. low–moderate quality evidence. 100% agreement. low–moderate quality evidence**.

FDG-PET/CT shows a high specificity of 92% and good sensitivity of 87% reported for detecting PM on a patient basis but does not appear to improve peritoneal staging nor change treatment over CT substantially [25, 26]. Adding PET to anatomical images can increase lesion conspicuity due to the higher contrast ratio of tracer uptake to the background. However, subcentimeter implants, particularly PM smaller than 5 mm, are inconsistently visualised owing to the problematic assessment of diffusely infiltrative deposits and insufficient tracer uptake in small-sized and mucinous lesions [27]. However, PET/CT can contribute by detecting metastases in normal-appearing lymph nodes. PET/CT can thus identify unexpected extra-abdominal nodal disease sites that affect resectability [28].

**6. Whole-body MRI and FDG-PET/CT can be a problem-solver in colorectal and ovarian cancer patients suspected of PM /recurrence. Both modalities can help detect other extraperitoneal metastases missed on prior imaging. 100% agreement, low–moderate quality evidence**.

Both PET/CT and WB-DWI/MRI have the advantage of high contrast resolution and have the ability to detect tumours, respectively, based on metabolic and microstructural properties without the need for underlying anatomical distortions facilitating the detection of subcentimeter mediastinal or cervical lymph nodes, pleural metastases and small liver metastases that do not cause parenchymal distortion or are hampered in their detection by underlying steatosis [29]. In ovarian cancer, liver and lung metastases are extremely rare at primary diagnosis, and distant metastatic disease mainly consists of supradiaphragmatic lymphadenopathy and pleural metastases, for both of which FDG-PET/CT and WB-DWI/MRI have been shown to outperform CT for their detection. In colorectal cancer patients with peritoneal metastases and candidates for HIPEC surgery, abdominal MRI with DWI had added value over CT in detecting abdominopelvic extraperitoneal metastases relevant to the treatment planning [24].

**7. An abdominal CT for PM consists of 1/1.5** **mm section images with 3** **mm axial and coronal or/and sagittal reconstructions in the portal venous phase. The use of positive or negative oral contrast agents is highly recommended but can be omitted based on local preferences. 85% agreement, low–moderate quality evidence**.

There is no relevant literature comparing different CT protocols for detecting PM. Therefore, the standard abdominal CT in the portal venous phase is recommended (Table 1). To be able to detect small-volume disease, 1/1.5 mm section images are advised. There was some debate in the guideline committee concerning using positive, negative, or no oral contrast agents. Most expert centres use positive oral contrast to improve the detection of PM near the bowels more easily.

**Table 1:** CT protocol parameters for staging peritoneal metastases

CT parameters	
Respiration phase	Single breath-hold
Tube voltage	< 120 KVp
Tube current	As suggested by the automatic exposure control
Slice thickness	< 0.75 mm, 1/1.5 mm reconstructed
Pitch	< 0.5 mm
Portal venous phase (ms)	60–80 s after contrast injection
Reconstruction algorithm	Soft tissue
Oral contrast	Positive or negative oral contrast is highly recommended

**8. An abdominal or whole-body 1.5- or 3-T MRI for PM consists of axial T2 (slice thickness** ≤ **4** **mm), STIR-DWI (b50 and b1000 s/mm**^**2**^**), and post-contrast (after 3–5** **min) T1 sequences in axial/coronal view. Axial pre-contrast T1 and coronal T2-weighted sequences are optional. One litre pineapple juice as a negative oral contrast and an intravenous antiperistatical agent is highly recommended but not obligatory. 85% agreement, low–moderate quality evidence (**Tables 2, 3**).**

**Table 2:** Example protocol for staging peritoneal metastases on a 1.5-T MRI

		STIR-DWI		T2 SSTSE/HASTE		Contrast-enhanced 3D GE	
		Transverse	Coronal	Transverse	Corona	Transverse	Coronal
Image stations abdomen-pelvis		4	MPR	2	3	3	1
Respiration		Free breathing		Respiratory	Free breathing	Breath-hold	Breath-hold
Fat suppression		STIR (TI = 160–180 ms)		None	None	m (DIXON)	m (DIXON)
b-values (s/mm²)		50–1000		NA	NA	NA	NA
Parallel imaging factor		2		2	2	2	2
Repetition time (TR) (ms)		6622		6622	6622	3.65	4.32
Echo time (TE) (ms)		58		80–87	80–87	1.77	1.98
Slice thickness (mm)		5	5	5	5	3	3
Slice number		43/station		41	38	112/104/88	60
Intersection gap (mm)		0		0.6	0.6	0	0
Field of view (FOW) (mm)		420 × 349		342 × 400	400 × 500	400 × 300	450 × 337
Acquired voxel size (mm)		3.36 × 3.36		1.4 × 1.6	1.56 × 1.56	1.79 × 1.25	2.01 × 1.41
Reconstructed voxel size (mm)		1.68 × 1.68		1 × 1	1.56 × 1.56	0.63 × 0.63	0.7 × 0.7
Number of signal averages (NSA)		1–3 (b1000)		1	1	1	1

*DWI* diffusion-weighted imaging, *STIR* short T1 inversion recovery, *SPAIR* spectrally adiabatic inversion recovery, *SSTSE* single-shot Turbo spin-echo, *HASTE* half-Fourier single-shot turbo spin echo, *NA* not applicable

**Table 3:** Protocol for staging peritoneal metastases on a 3-T MRI

	STIR-DWI		T2 SSTSE/HASTE		Contrast-enhanced 3D GE	
	Transverse	Coronal	Transverse	Coronal	Transverse	Coronal
Image stations abdomen-pelvis	4	MPR	2	3	3	1
Respiration	Free breathing		Respiratory triggered	Free breathing	Breath-hold	Breath-hold
Fat suppression	STIR (TI = 250 ms)		None	None	m (DIXON)	m (DIXON)
b-values (s/mm²)	50–1000		NA	NA	NA	NA
Parallel imaging factor	2.5		2	4	2	2
Repetition time (TR) (ms)	8454		30,000	3000	3.6	3.2
Echo time (TE) (ms)	67		87	87	1.25–2.20	1.25–2.20
Slice thickness (mm)	5	5	6	6	1.5	1.5
Slice number	50/station		41	35	90	133
Intersection gap (mm)	0.1		0.6	0.6	0	0
Field of view (FOW) (mm)	420 × 329		357 × 339	375 × 447	375 × 304	400 × 352
Acquired voxel size (mm)	4.57 × 4.71		1 × 1	1 × 1	1.49 × 1.5	1.49 × 1.5
Reconstructed voxel size (mm)	2.19 × 2.16		0.78 × 0.78	0.93 × 0.93	0.71 × 0.71	0.71 × 0.71
Number of signal averages (NSA)	1–3 (b1000)		1	1	1	1

*DWI* diffusion-weighted imaging, *STIR* short T1 inversion recovery, *SPAIR* spectrally adiabatic inversion recovery, *SSTSE* single-shot Turbo spin-echo, *HASTE* half-Fourier single-shot turbo spin echo, *NA* not applicable

DWI detects tumours by combining heavy diffusion weighting and background signal suppression of organs and ascites. Applying a sufficiently high b-value (e.g., b1000) allows perceiving small water molecule movements, thereby maximising the information provided by the molecule movements in the extravascular extracellular space and optimising the detection of restricted diffusion in tumours as opposed to benign or background tissue. The DWI sequence can be further optimised for large-volume body imaging and lesion detection by using a short T1 inversion recovery (STIR) instead of the standard spectral attenuated inversion recovery (SPAIR) prepulse. The suppressive effect of the STIR prepulse on the bowel wall, having a short T1 value, facilitates the detection of serosal metastases. At the same time, it is more robust to inhomogeneity-induced fat suppression and susceptibility artefacts [30]. T2 and post-contrast T1 weighted sequences primarily act to locate lesions detected by DWI anatomically, correlate physiologically impeded diffusion attributable to T2 shine-through or anatomical structures, and facilitate the detection of lesions below the spatial resolution limit of DWI (< 4 mm). For this purpose, delayed Gadolinium-enhanced T1 images obtained 3–5 min after contrast injection show the highest accuracy in depicting peritoneal tumour deposits due to their slow accumulation of contrast [31].

Combining a negative peroral contrast and an intravenous antispasmodic can improve the detection of small serosal deposits by reducing physiological bowel wall hyperintensity and susceptibility artefacts caused by intraluminal air, which may obscure or be mistaken for serosal deposits [32]. Therefore, it is recommended that patients drink 1 L pineapple juice, starting 1–1.5 h before the examination. Pineapple juice contains manganese, which will optimise the DWI images. If pineapple juice is contra-indicated (e.g., diabetes, chronic renal failure), 7% Barium diluted in 1 L of water is a reasonable alternative. However, some expert centres find that not all patients are able or willing to drink 1 L of pineapple juice. This entails that patients (and technicians) must be motivated to comply.

**9. FDG PET/CT for PM should be performed according to the ‘EANM procedure guidelines for tumour imaging: version 2’. The combination with a CT of diagnostic quality, as described in statement 7, is highly recommended. 100% agreement, low–moderate quality evidence**.

The guideline committee refers to **‘**EANM procedure guidelines for tumour imaging: version 2’ for the FDG PET/CT imaging protocol for PM in colorectal and ovarian cancer [33].

**10. There are no differences in FDG PET/CT, CT, or MR imaging protocols of the abdomen for PM between colorectal and ovarian cancer. 100% agreement, low–moderate quality evidence**.

Current literature and expert opinion do not advocate different imaging protocols.

**11. The imaging report of colorectal cancer patients should include the extent of PM using the Peritoneal Cancer Index (PCI) combined with a qualitative description of the involvement of surgically critical sites and involved abdominal organs. Tools like PROMISE and PAUSE can be used to optimise and standardise the imaging report. 100% agreement, low–moderate quality evidence**.

A descriptive report remains the mainstay in reporting peritoneal metastases. However, a more standardised scoring system is advised for the location and extent of the PM. Especially MRI can accurately predict PCI in colorectal and ovarian cancer patients. In colorectal cancer, the PCI is widely validated and used by clinicians. Therefore, it is logical to adopt this scoring system in the radiological report. However, some multidisciplinary teams might prefer to use PROMISE or PAUSE [34, 35]. The PeRitOneal MalIgnancy Stage Evaluation (PROMISE) is an internet application which automatically calculates PCI and other surgically validated scores such as Gilly score, SPCI, Fagotti, and Fagotti-modified scores. The acronym PAUSE is used to emphasise the key imaging features that a radiology report should include like; P, primary tumour and peritoneal carcinomatosis index (PCI) as estimated by imaging; A, ascites and abdominal wall involvement; U, unfavourable sites of involvement; S, small bowel and mesenteric disease; E, extraperitoneal metastases. Radiologists should be in close contact with their clinicians to optimise their reports to local preferences. Structured reporting can be of added value for both radiological and clinicians and should be contemplated if technically feasible.

**12. The imaging report of ovarian cancer patients must include the extent of PM using one quantitative scoring method of local preference in combination with a qualitative description of the involvement of surgically critical sites and involved abdominal organs. Tools like PROMISE and PAUSE can be used to optimise and standardise the imaging report. 100% agreement, low–moderate quality evidence**.

Similar to reporting in colorectal cancer, a descriptive report concerning the location and extent of the PM, a more standardised scoring system is recommended. Although MRI can accurately predict the PCI and involvement of surgically critical sites in ovarian cancer patients, it is not yet widely used in ovarian cancer. Therefore, local preferences should dictate which standardised scoring system is to be used by the multidisciplinary team, including the radiologists. Radiologists should be actively involved in the MDT, and their reports should be tailored to local preferences.

Structured reporting can be of added value for both radiological and clinicians and should be contemplated if technically feasible.

**13. Preferably, all colorectal and ovarian cancer patients with PM should be discussed in a multidisciplinary tumour board, including a radiologist with experience in imaging of peritoneal metastases. 100% agreement, low–moderate quality evidence**.

Multidisciplinary tumour boards (MDTs) are the cornerstone in the treatment of oncological patients [36, 37]. MDTs have been shown to improve patient outcomes [38]. Close collaboration of all involved professions and disciplines improves cancer care. The radiologist plays an important role in the MDT and should stage the disease correctly and demonstrate to the clinicians the location and extent of the disease to optimise surgical planning. Potential complex surgical sites like the liver hilum, diaphragm, mesentery, and pelvis should be demonstrated during the MDT. This means that a dedicated radiologist must be present at the MDT to be able to address and demonstrate all important imaging findings.

### Limitations

A lack of randomised data concerning the imaging of PM hampers making evidence-based guidelines. However, systematic reviews and large observational studies were used to formulate the recommendations. The guideline committee encourages further research involving both the development and clinical integration of existing and novel imaging techniques. Another limitation is that the preferences of the patients are not addressed in the current guidelines.

### Implementation and adherence to guidelines

In the daily clinic, CT can be used for initial screening when the clinical diagnosis of peritoneal metastases is unclear or when an extensive disease load not amenable to surgery is suspected. If upfront operability assessment is required, or when there are equivocal CT findings, DW-MRI is the preferred method for peritoneal staging and simultaneously allows for the detection of extraperitoneal disease. FDG-PET/CT can be used to detect lymphadenopathy and extraperitoneal metastases.

The committee aimed to make the guidelines applicable to hospitals throughout Europe. However, the committee is aware that not all hospitals have implemented MRI in the daily diagnostic workup of ovarian and colorectal cancer patients with PM. This entails that large implementation studies must be performed to implement MRI in the diagnostic workup to improve adherence to this guideline further. By doing so, all patients with advanced colorectal and ovarian cancer can receive the most optimal diagnostic workup to guide their tailored treatment.

### Updating the guideline

These guidelines should be updated in or before 2028 if new evidence of good quality warrants a significant modification to these recommendations.

## Supplementary information

ELECTRONIC SUPPLEMENTARY MATERIAL

## Abbreviations

CT: Computed tomography
DWI: Diffusion-weighted imaging
EANM: European Association of Nuclear Medicine
ESGAR: European Society of Gastrointestinal and Abdominal Radiology
ESUR: European Society of Urogenital Radiology Evaluation
FDG: Fluorine-18-deoxyglucose
GRADE: Grading of Recommendations Assessment, Development and Evaluation
HIPEC: Hyperthermic intraperitoneal chemotherapy
MDT: Multidisciplinary tumour boards
MRI: Magnetic resonance imaging
PAUSE: P, primary tumour and peritoneal carcinomatosis index (PCI) as estimated by imaging; A, ascites and abdominal wall involvement; U, unfavourable sites of involvement; S, small bowel and mesenteric disease; E, extraperitoneal metastases
PCI: Peritoneal Cancer Index
PET/CT: Positron emission tomography/computed tomography
PM: Peritoneal metastases
PROMISE: Peritoneal malignancy stage evaluation
PSOGI: Peritoneal Surface Oncology Group International
SPAIR: Spectral attenuated inversion recovery
STIR: Short T1 inversion recovery
WB: Whole body
