# Supplementary material for: Imaging of peritoneal metastases of ovarian and colorectal cancer: joint recommendations of ESGAR, ESUR, PSOGI, and EANM
Source: Eur Radiol. 2024 Nov 5;35(5):2712–22. doi: 10.1007/s00330-024-11124-5 (PMC12021955; doi:10.1007/s00330-024-11124-5)
Supplement: Supplementary file 1 — ELECTRONIC SUPPLEMENTARY MATERIAL [file 330_2024_11124_MOESM1_ESM.pdf]

Imaging of peritoneal metastases of ovarian and colorectal cancer: joint recommendations of ESGAR, ESUR, PSOGI, and EANM ELECTRONIC SUPPLEMENTARY MATERIAL

Appendix A.

Medline

((("Peritoneal Neoplasms"/ and exp "Neoplasm Metastasis"/) or "Peritoneal Neoplasms"/sc or ((peritoneal* or peritoneum) adj5 (metastas* or carcinomatos* or secondary or seeding or implant* or dissemination or malignan* or "surface disease*" or staging or infiltrat* or disseminat*) or "peritoneal cancer index").ti,ab,kf.) and (exp "ovarian neoplasms"/ OR ((ovary OR ovaries OR ovarian OR ovarium OR ovaria OR ovaric OR ovarii OR "corpus luteum" OR "corpora lutea" OR adnex*) ADJ4 (neoplas* OR tumor* OR tumour* OR cancer* OR malign* OR oncolog* OR carcinom* OR neoplasm* OR lymphoma* OR sarcoma* OR cyst* or metastas* or adenocarcinom*)).ti,ab,kf. OR exp "Colorectal Neoplasms"/ OR ((colorectal or adenomatous or colon* or sigmoid or rectal or rectum or anus or anal or coloanal) ADJ4 (neoplas* OR tumor* OR tumour* OR cancer* OR malign* OR oncolog* OR carcinom* OR neoplasm* or metastas* or polyp* or lesion*)).ti,ab,kf.) and (((exp "Magnetic Resonance Imaging"/ or (MRI or MRIs* or fMRI* or (("magnetic resonance" or "magnetization transfer" or nmr or mr or "proton spin" or "chemical shift" or "spin echo" or "transfer contrast") adj1 (imag* or tomograph*))).ti,ab,kf.) OR (exp "Tomography, X-Ray Computed"/ or (((compute* or "computer assist*" or "proton emission" or "positron emission") adj3 (tomograph*)) or tomodensitometr* or "electron beam tomograph*" or ((PET) adj1 (CT)) or CECT or MDCT or ((CAT or CT or PET) adj1 (scan* or X-ray*))).ti,ab,kf.)))) or ((("Peritoneal Neoplasms"/ and exp "Neoplasm Metastasis"/) or "Peritoneal Neoplasms"/sc or ((peritoneal* or peritoneum) adj5 (metastas* or carcinomatos* or secondary or seeding or implant* or dissemination or malignan* or "surface disease*" or staging or infiltrat* or disseminat*) or "peritoneal cancer index").ti,ab,kf.) and (((exp "Magnetic Resonance Imaging"/ or (MRI or MRIs* or fMRI* or (("magnetic resonance" or "magnetization transfer" or nmr or mr or "proton spin" or "chemical shift" or "spin echo" or "transfer contrast") adj1 (imag* or tomograph*))).ti,ab,kf.) OR (exp "Tomography, X-Ray Computed"/ or (((compute* or "computer assist*" or "proton emission" or "positron emission") adj3 (tomograph*)) or tomodensitometr* or "electron beam tomograph*" or ((PET) adj1 (CT)) or CECT or MDCT or ((CAT or CT or PET) adj1 (scan* or X-ray*))).ti,ab,kf.))) and (exp "Sensitivity and Specificity"/ or exp "Predictive value of Tests"/ or (accura* or predict* or sensitiv* or specific* or staging or prognos* or "interobserver agreement*" or detect* or evaluat* or reliab* or AUC or "area under the curve" or "diagnostic perform*").ti,ab,kf.))

Embase.com

((('peritoneum tumor'/exp and 'metastasis'/exp) or ((peritoneal* or peritoneum) NEAR/5 (metastas* or carcinomatos* or secondary or seeding or implant* or dissemination or malignan* or "surface disease*" or staging or infiltrat* or disseminat*) or "peritoneal cancer index"):ti,ab,kw) and ('ovary tumor'/exp OR ((ovary OR ovaries OR ovarian OR ovarium OR ovaria OR ovaric OR ovarii OR "corpus luteum" OR "corpora lutea" OR adnex*) NEAR/4 (neoplas* OR tumor* OR tumour* OR cancer* OR malign* OR oncolog* OR carcinom* OR neoplasm* OR lymphoma* OR sarcoma* OR cyst* or metastas* or adenocarcinom*)):ti,ab,kw OR exp 'colorectal cancer'/exp OR ((colorectal or adenomatous or colon* or sigmoid or rectal or rectum or anus or anal or coloanal) NEAR/4 (neoplas* OR tumor* OR tumour* OR cancer* OR malign* OR oncolog* OR carcinom* OR neoplasm* or

metastas* or polyp* or lesion*)):ti,ab,kw) and ((('nuclear magnetic resonance imaging'/exp or (MRI or MRIs* or fMRI* or (("magnetic resonance" or "magnetization transfer" or nmr or mr or "proton spin" or "chemical shift" or "spin echo" or "transfer contrast") NEAR/1 (imag* or tomograph*))):ti,ab,kw) OR ('x-ray computed tomography'/exp or 'positron emission tomography'/exp or (((compute* or "computer assist*" or "proton emission" or "positron emission") NEAR/3 (tomograph*)) or tomodensitometr* or "electron beam tomograph*" or ((PET) NEAR/1 (CT)) or CECT or MDCT or ((CAT or CT or PET) NEAR/1 (scan* or X-ray*))):ti,ab,kw)))) or ((('peritoneum tumor'/exp and 'metastasis'/exp) or ((peritoneal* or peritoneum) NEAR/5 (metastas* or carcinomatos* or secondary or seeding or implant* or dissemination or malignan* or "surface disease*" or staging or infiltrat* or disseminat*) or "peritoneal cancer index"):ti,ab,kw) and ((('nuclear magnetic resonance imaging'/exp or (MRI or MRIs* or fMRI* or (("magnetic resonance" or "magnetization transfer" or nmr or mr or "proton spin" or "chemical shift" or "spin echo" or "transfer contrast") NEAR/1 (imag* or tomograph*))):ti,ab,kw) OR ('x-ray computed tomography'/exp or 'positron emission tomography'/exp or (((compute* or "computer assist*" or "proton emission" or "positron emission") NEAR/3 (tomograph*)) or tomodensitometr* or "electron beam tomograph*" or ((PET) NEAR/1 (CT)) or CECT or MDCT or ((CAT or CT or PET) NEAR/1 (scan* or X-ray*))):ti,ab,kw))) and ('diagnostic accuracy'/exp or 'sensitivity and specificity'/exp or 'predictive value'/exp or (accura* or predict* or sensitiv* or specific* or staging or prognos* or "interobserver agreement*" or detect* or evaluat* or reliab* or AUC or "area under the curve" or "diagnostic perform*"):ti,ab,kw))

Scopus

((TITLE-ABS-KEY((peritoneal* or peritoneum) W/5 (metastas* or carcinomatos* or secondary or seeding or implant* or dissemination or malignan* or "surface disease*" or staging or infiltrat* or disseminat*) or "peritoneal cancer index")) AND (TITLE-ABS-KEY((ovary OR ovaries OR ovarian OR ovarium OR ovaria OR ovaric OR ovarii OR "corpus luteum" OR "corpora lutea" OR adnex*) W/4 (neoplas* OR tumor* OR tumour* OR cancer* OR malign* OR oncolog* OR carcinom* OR neoplasm* OR lymphoma* OR sarcoma* OR cyst* or metastas* or adenocarcinom*)) OR TITLE-ABS- KEY((colorectal or adenomatous or colon* or sigmoid or rectal or rectum or anus or anal or coloanal) W/4 (neoplas* OR tumor* OR tumour* OR cancer* OR malign* OR oncolog* OR carcinom* OR neoplasm* or metastas* or polyp* or lesion*))) AND (TITLE-ABS-KEY(MRI or MRIs* or fMRI* or (("magnetic resonance" or "magnetization transfer" or nmr or mr or "proton spin" or "chemical shift" or "spin echo" or "transfer contrast") W/1 (imag* or tomograph*)) OR ((compute* or "computer assist*" or "proton emission" or "positron emission") W/3 (tomograph*)) or tomodensitometr* or "electron beam tomograph*" or ((PET) W/1 (CT)) or CECT or MDCT or ((CAT or CT or PET) W/1 (scan* or X-ray*))))) OR ((TITLE-ABS-KEY((peritoneal* or peritoneum) W/5 (metastas* or carcinomatos* or secondary or seeding or implant* or dissemination or malignan* or "surface disease*" or staging or infiltrat* or disseminat*) or "peritoneal cancer index")) AND (TITLE-ABS-KEY(MRI or MRIs* or fMRI* or (("magnetic resonance" or "magnetization transfer" or nmr or mr or "proton spin" or "chemical shift" or "spin echo" or "transfer contrast") W/1 (imag* or tomograph*)) OR ((compute* or "computer assist*" or "proton emission" or "positron emission") W/3 (tomograph*)) or tomodensitometr* or "electron beam tomograph*" or ((PET) W/1 (CT)) or CECT or MDCT or ((CAT or CT or PET) W/1 (scan* or X-ray*)))) AND (TITLE-ABS-KEY(accura* or predict* or sensitiv* or specific* or staging or prognos* or "interobserver agreement*" or detect* or evaluat* or reliab* or AUC or "area under the curve" or "diagnostic perform*")))
